# The Effect of Cold Press Chia Seed Oil By-Products on the Rheological, Microstructural, Thermal, and Sensory Properties of Low-Fat Ice Cream

**DOI:** 10.3390/foods10102302

**Published:** 2021-09-28

**Authors:** Ilker Atik, Zeynep Hazal Tekin Cakmak, Esra Avcı, Salih Karasu

**Affiliations:** 1 Food Technology Program, Afyon Vocational School, Afyon Kocatepe University, Afyonkarahisar 03200, Turkey; ilkeratik@hotmail.com; 2 Department of Food Engineering, Faculty of Chemical and Metallurgical Engineering, Davutpasa Campus, Yildiz Technical University, Istanbul 34210, Turkey; zhazaltekin@gmail.com (Z.H.T.C.); esravci93@hotmail.com (E.A.)

**Keywords:** by-product, rheological properties, optimization, melting profile

## Abstract

This study aimed to investigate the utilization of cold-pressed chia-seed oil by-products (CSOB) in a low-fat ice cream formulation as a fat replacer and stabilizer. In the study, ice cream emulsion mixtures were formulated by using 0.2–0.4% xanthan gum (XG), 2.5–12.5% fat, and 1–3% CSOB. Optimization was performed using the response surface methodology (RSM) and full factorial central composite design (CCD) based on the flow behavior rheological properties of the emulsions obtained from 17 different experimental points. All of the emulsion samples showed non-Newtonian shear-thinning flow behavior. The consistency coefficient (*Κ*) values of the emulsion samples were found to be 4.01–26.05 Pas^n^ and were significantly affected by optimization parameters (*p* < 0.05). The optimum formulation was determined as 0.29% XG, 2.5% CSOB, 2.5% fat. The low-fat (LF-IC) and full-fat control samples (FF-IC) were compared to samples produced with an optimum formulation (CBLF-IC) based on the steady shear, frequency sweep, and 3-ITT (three interval thixotropy test) rheological properties, thermal properties, emulsion stability, light microscope images, and sensory quality. CBLF-IC showed similar rheological behavior to FF-IC. The mix of CBLF-IC showed higher emulsion stability and lower poly-dispersity index (PDI) value and fat globule diameters than those of FF-IC and LF-IC. The thermal properties of the samples were significantly affected by the addition of CSOB in an ice cream mix. CBLF-IC exhibited a lower temperature range (ΔT), enthalpy of fusion (ΔH_f_), and freezing point temperature (T_f_) than those of FF-IC and LF-IC. While CBLF-IC exhibited a higher overrun value than other samples, it showed similar sensory properties to the FF-IC sample. The results of this study suggested that CSOB could be used successfully in low-fat ice cream production. This study also has the potential to gain new perspectives for the evaluation of CSOB as a fat substitute in a low-fat ice cream.

## 1. Introduction

Ice cream is a frozen milk-based dessert as a frozen aerated emulsion (O/W) containing partially combined fat globules, air bubbles, and ice crystals. Ice cream includes milk, milk cream, emulsifiers, sweeteners, stabilizers, and flavorings [1]. Ice cream generally contains high dairy or non-dairy fat (10–16%) [2,3]. As one of the most important ingredients in ice cream, milk fat interacts with other ingredients to improve texture, mouthfeel, creaminess, and overall sensation of lubricity [4]. Ice cream is a popular dessert, especially during hot weather, with an annual global consumption of around 2 L per person [5]. However, the demand for low-calorie foods has increased in recent years. Therefore, many studies have been carried out to develop new additives and new products in the food industry to meet this demand. In order to ensure the stability of ice cream, some special additives with stabilizer and emulsifier properties are required, and these additives are used in ice cream production.

Stabilizers are preferred in ice cream production because they provide specific and important functions such as increasing the viscosity and smoothness of the ice cream mix, improving aeration, reducing ice recrystallization, and the rate of structural collapse during melting [2,6]. Emulsifiers are substances that provide a fine dispersion of foods by reducing surface tension [7]. Emulsifiers minimize the formation of ice crystals by increasing the volume capacity of the ice cream and its resistance to melting. Stabilizers and emulsifiers also provide the ice cream with dryness and hardness, as well as a smooth structure and the desired oily feeling [8,9,10].

Chia (*Salvia hispanica* L.) seed, belonging to the *Lamiaceae* family, has a high amount of oil (25–38%), carbohydrates (26–41%), high dietary fiber (18–30%), and protein (19–23%) [11]. Chia seeds have emulsifying and stabilizing properties due to their high protein content and water-soluble branching polysaccharides. They have the potential to be used as a natural emulsifier and stabilizer in many food compositions [12,13]. Researchers have studied the effect of chia seed gel mucilage on ice cream as a stabilizer and emulsifier [14,15,16,17,18]. In our study, different from these studies, a cold press oil by-product was used in the preparation of ice cream. In addition, in our study, the by-product ratio was determined under optimum conditions, and comprehensive properties such as rheological, microstructural, emulsion stability, thermal and sensory properties in the low-fat and high-fat control samples were analyzed in this study. It was also used for the first time in this study for 3-ITT analysis and thermal loop rheology of ice cream samples. Our research paper contains differences from other studies both in terms of material and analysis methods.

Fat substitutes can be used to reduce the amount of fat in foods and replace it with oil-like substances and reduce the caloric value with new additives. Fat substitutes can provide many of the properties that fat gives to foods and be classified as carbohydrate-derived fat substitutes, protein-derived fat substitutes, synthetic fat substitutes, and fat compound substitutes. When these substances are used instead of fat in foods, the fat in the food can be partially or completely reduced, and the energy from fat is minimized but provides palatable products [19]. Recently, cold-pressed oil industry by-products have also been used as fat substitutes [20,21,22]. Cold-press oil by-products such as cold-press oils include a high amount of protein, carbohydrate, fiber contents, and nutritive components without any solvent trace, and these by-products can be used as a natural fat replacer, emulsifier, stabilizer, and a natural antioxidant source in food emulsions [23,24].

Chia-seed by-products derived from the cold-press oil industry have the potential to be utilized as a building strengthener and fat replacer due to their ability to develop relatively viscous solutions. Furthermore, Capitani et al. [25] reported that the residual meal/by-product obtained from the oil-extracting process of chia seeds was determined as a good source of total dietary fiber, consisting mostly of insoluble dietary fiber. Therefore, chia seed by-products can improve food texture, utilize a role as texturing and stabilizing agent due to high dietary fiber content. Akcicek and Karasu [20] used the cold-pressed chia seed oil by-products as a fat replacer in a low-fat salad dressing. However, chia seed oil by-products as a fat replacer, stabilizer, and emulsifier in a low-fat ice-cream formulation have not been investigated. Many studies are present in the literature about the development of low-fat ice cream formulations [7,26,27,28,29,30].

The main purpose of this study is to produce ice cream with an optimum feature of low-fat content by using cold-pressed chia seed oil by-products without causing any change in the rheological and melting properties of ice cream. In this study, the optimization process was carried out based on the rheological analysis results of the ice creams produced with different formulations. The rheological and melting properties of the ice creams produced after the optimization process were investigated. Thus, production parameters with optimum rheological and melting properties were determined.

## 2. Material and Methods

### 2.1. Material

In this study, CSOB, xanthan gum (XG), egg yolk powder (EYP), milk cream (35% milkfat), pasteurized milk (1.5% milk fat), sugar, and water were used for producing ice cream samples. CSOB, 8.07% fat, 56.10% total carbohydrate including fiber, 23.50% protein, 29.26% fiber, 5.21% ash, 6.85% moisture, was supplied from ONEVA Food Co. (Istanbul, Turkey). XG and egg yolk powder (EYP) were obtained from Sigma-Aldrich (Sigma Chemical Co., St. Louis, MO, USA), and the sugar, milk, and milk cream were purchased from the local market.

### 2.2. Methods

The study was carried out in two stages. In the first stage, the responses were obtained at 17 different points for ice cream samples through the Design Expert Software (Version 7; Stat-Easy Co., Minneapolis, MN, USA), and the formulation was optimized according to flow behavior characteristics. In the second stage, the dynamic rheological and melting profile properties of the ice cream samples produced according to the optimum formulation were examined and characterized.

#### 2.2.1. Experimental Design

The formulation of ice cream samples was determined using the Design Expert Software (Version 7; Stat-Easy Co., Minneapolis, MN, USA). Response surface methodology (RSM) was carried out for the study of three factors, namely fat concentration (2.5–12.5 g/100 g) (*X*_1_), CSOB concentration (1–3 g/100 g) (*X*_2_), and gum concentration (0.2–04 g/100 g) (*X*_3_), using an unblocked full factorial central composite design (CCD). Seventeen different experimental points were obtained by using Design Expert Software (Version 7; Stat-Easy Co., Minneapolis, MN, USA) to determine the optimum amount of fat, CSOB, and gum content. For the estimation of the error, the design consists of three of the factorial points. A quadratic model was fitted to the experimental data for each response. Model applicability was evaluated based on the *R*^2^, *R*^2^-adj, lack of fit, F, and *p*-values obtained from ANOVA. Response surface plots showing the effect of model parameters on the *K* value were built by Design Expert Software. The optimization was performed based on the highest desirability value. The formulation with the lowest fat content with a desirability value of 1 was determined as the optimum formulation. Three central points were used. Analysis of all points was performed in triplicate, and the results were reported as mean values and standard deviations. The formulation of the samples has been determined in accordance with the ice cream standard. Accordingly, 2.5% fat ratio representing low-fat ice cream (0.5 ≤ milk fat < 3), 7.5% fat ratio representing semi-fat ice cream (3 ≤ milk fat < 8), and 12.5% fat ratio representing fat ice cream (8 ≤ milk fat ≤ 13) were used. Based on these fat ratios, the amount of milk and milk cream to be used has been calculated. The percentage of fat, XG, and CSOB vary in formulations, and the amounts of sugar (12%) and EYP (3%) remain constant. Seventeen formulations obtained in this way are shown in Table 1.

#### 2.2.2. Ice-Cream Preparation

Ice cream formulations consisted of 2.5–12.5% fat (derived from 1.5% milk fat and 98.5% milk cream), 12% sugar, 3% egg yolk powder (EYP) and 0.2–0.4% xanthan gum (XG), 1–3% CSOB. XG and milk were weighed according to the formulations determined in the first stage of ice cream production. XG was slowly added in order to dissolve it thoroughly and kept in a magnetic stirrer at 1000 rpm. Then, CSOB, which was weighted according to the formulation, was added and mixed. Then milk cream was added sequentially. The EYP and sugar were added and mixed. Then, the samples were homogenized with ultra turrax (Daihan. HG-15D) at 1000 rpm for 3 min. After this process, the ice cream mixture samples were kept at 0–4 °C for 2 days to mature. Afterward, ice cream was produced from the mixes using an ice cream machine (DeLonghi IL Gelataio ICK5000, Treviso, Italy). Ice cream samples were stored at −18 °C for analysis.

#### 2.2.3. Analysis of the Ice Cream Mix

##### Rheological Analyzes

Flow behavior, dynamic, and 3-ITT rheological properties of ice cream mixes were determined using a stress and temperature-controlled rheometer (MCR 302, Anton Paar, Australia). A parallel plate probe (PP50, Anton Paar, Australia) was used for rheological measurement. All measurements were performed at 25 °C and duplicated for the accuracy of the results.

##### Flow Behavior Rheological Properties

Flow behavior rheological properties of ice cream samples prepared using CSOB were determined using a parallel plate probe (plate diameter 50 mm, gap size 0.5 mm) with a shear rate in the range 0.1–100 (s^−1^). The measurement was carried out at a constant temperature of 25 °C, and 3 parallel studies were carried out for each sample. The data obtained from the rheological analysis were fitted to the power law model, and nonlinear regression was used to calculate model parameters;

(1) $$\tau =K\gamma^{n}$$

In Equation (1), the *τ* value represents the shear stress (Pa), *K* the consistency coefficient (Pas^n^), *γ* the shear rate (s^−1^), and *n* the flow behavior index.

##### Dynamic Rheological Properties

Parallel plate configuration was used for the dynamic rheological analysis of ice cream samples. Initially, the amplitude sweep test was performed between 0.1% and 100% strain to determine the linear viscoelastic region, and according to the result, the frequency sweep test was studied in the frequency range of 0.1–10 Hz and angular velocity of 0.1–64 s^−1^ (ω). Elastic modulus ($G^{\prime }$) and viscose modulus ($G^{\prime \prime }$) corresponding to angular velocity and frequency values were determined. The parameters for dynamic rheological properties were found using the power law model and nonlinear regression;

(2) $$G^{\prime }=K^{\prime }{\left(\omega \right)}^{n^{\prime }}$$

(3) $$G^{\prime \prime }=K^{\prime \prime }{\left(\omega \right)}^{n^{\prime \prime }}$$

In Equations (2) and (3), the $G^{\prime }$ value represents storage modulus (Pa), $G^{\prime \prime }$ value loss modulus (Pa), ωangular velocity value (s^−1^), $K^{\prime }$, $K^{\prime \prime }$ consistency coefficient values (Pas^n^) and $n^{\prime }$, $n^{\prime \prime }$ flow behavior index values.

##### 3-ITT

3-ITT rheological properties of ice cream samples that contained CSOB were determined as 0.5 s^−1^ as constant shear rate value and 150 s^−1^ as variable shear rate value. The linear viscoelastic region has been taken into consideration in the selection of the values, and the linear viscoelastic region of the samples ends at 50 s^−1^. The ice cream samples were subjected to a very low shear rate (0.5 s^−1^) for 100 s during the first time interval. In the second time interval, 150 s^−1^ was exposed to the determined cutting force for 40 s. In the third time interval, the dynamic rheological behavior of the ice cream in the second time interval was tested by subjecting the samples to the low shear rate level in the first time interval. For this purpose, the change in the viscoelastic solid structure ($G^{\prime }$) of the samples was observed. The behavior of samples produced using CSOB in the third time interval was modeled using a second-order structural kinetic model;

(4) $${\left[\frac{G^{\prime }-G_{e}}{G_{0}-G_{e}}\right]}^{1-n}=\left(n-1\right)kt-1$$

In the model, the $G^{\prime }$ value indicates the change in the storage module (Pa), $G_{0}$ indicates the initial storage module value (Pa) in the 3rd time interval, $G_{e}$ represents the storage module at the moment when the product fully recovered, in other words, the storage module (Pa) at the moment when the product is fully balanced, and *k* is the thixotropic velocity constant.

##### Emulsion Stability Test

The emulsion stability of ice cream mix was determined by the thermal loop test previously described by Tekin et al. [31]. The ice cream mix samples were subjected to ten thermal cycles from 23 to 45 °C in a high-temperature stability test. An angular frequency (ω) and strain value were 10 Hz and 0.5%, respectively. The heating and cooling rates were set at 11 °C/min. The maximum points of all cycles were recorded by using the rheometer software (Rheoplus for MCR 301) using the internal loop. The relative structural change value (Δ) was calculated for G* values by dividing the maximum value of each cycle by the value of the first cycle.

##### The Determination Particle Size

The size of the fat globules and zeta potential value of the samples was determined by the particle size measuring device (Nanosizer, Malvern Instruments, Worcestershire, UK). The samples were diluted 500-fold with ultrapure water before homogenization by stirring in an ultrasonic water bath for 1 min. The particle size of the samples was determined according to the dynamic light scattering technique [31].

#### 2.2.4. Analysis of the Ice Cream Samples

##### Overrun Measurement

The volumes of ice cream mixtures (just before freezing) and ice cream samples (immediately after freezing) were weighed, and values were recorded [15]. Overrun values of the ice cream samples were calculated according to the formula described by Equation (5);

(5) $$\mathrm{Overrun}\;(\%)=\frac{W_{2}-W_{1}}{W_{1}}\times 100$$

where *W*_1_, and *W*_2_ represent the weight of a unit volume of ice cream mix and the weight of a unit volume of ice cream after freezing, respectively.

##### Thermal Properties of Ice Cream Samples

The thermal properties of ice cream samples were analyzed by a differential scanning calorimeter (DSC) by A DTA-DSC (differential scanning calorimetry) operating at atmospheric pressure (STA44gf3, Netzsch, Germany) according to the method reported by Hwang et al. [32]. Ice cream samples of 10 mg were placed in a pre-weighed aluminum sample pan, the pan was sealed using a Quick Press pan crimper (T_zero_), and the thermal data were recorded from −20 to +50 °C in a nitrogen atmosphere with a heating rate of 1 °C/min. An empty pan was used as the reference. The flow rates of nitrogen gas for cooling were 50 mL/min. The onset temperatures (T_0_), T_f_, and ΔH_f_ of the transitions of ice formation and ice melting were determined. The onset temperatures are considered as the intersection of the tangent and baseline to the left side of the melting peak. Freezing points were determined by using the temperature of the steepest slope. The enthalpy of fusion was calculated by extrapolating the baseline under the peak by connecting the flat baseline before and after the melting peak and integrating the peak above the baseline. The amount of ice formed per gram of sample (freezable water) was determined by the method described by Soukoulis et al. [33] by dividing the melting enthalpy with the pure ice fusion latent heat (S = 334 J/g).

##### Sensory Analysis of Ice Cream

A trained panel of 10 members (graduate students and academic staff from the Food Engineering Department at Yildiz Technical University in Istanbul, Turkey) assessed the sensory characteristics of ice cream samples. Panel members were instructed on the ice cream samples prior to the commencement of the tests. The training consisted of a two-hour thorough presentation to the panelists on the purpose of the study and the characteristics of the samples. The panelists were asked to identify the optimal persimmon content for the required enhanced ice cream in terms of look and color, odor, taste and flavor, texture, melting resistance, and overall acceptability. Ice cream samples were evaluated using a scaling method of descriptive attributes for all parameters (1 = undesired, 9 = desired) [34].

##### Color Measurement

The color parameters of ice cream samples were measured using a colorimeter (CR-400 Konica, Minolta, Tokyo, Japan). The CIELAB coordinate system was used, and the L*, a* and b* parameters were evaluated. L*, a* and b* parameters represent whiteness/darkness, redness/greenness, and yellowness/blueness, respectively.

### 2.3. Statistical Analysis

Ice cream samples were produced in three replications, and three parallel measurements were performed from each replication. The mean and standard deviation values were presented. Statistical applications were carried out with the Statistica software package (Stat Soft Inc., Tulsa, UK). The Duncan multiple comparison test was used to compare the mean values of the parameters after optimization (*p* < 0.05). As a result of the applied rheological analysis, power law model parameters were calculated with the help of nonlinear regression analysis. The Statistica software program (Stat Soft Inc., Tulsa, UK) was used for nonlinear regression analysis.

## 3. Result and Discussion

### 3.1. Determination of Steady Shear Rheological Properties of Ice Cream Mix for the Formulation Optimization

**Figure 1:**
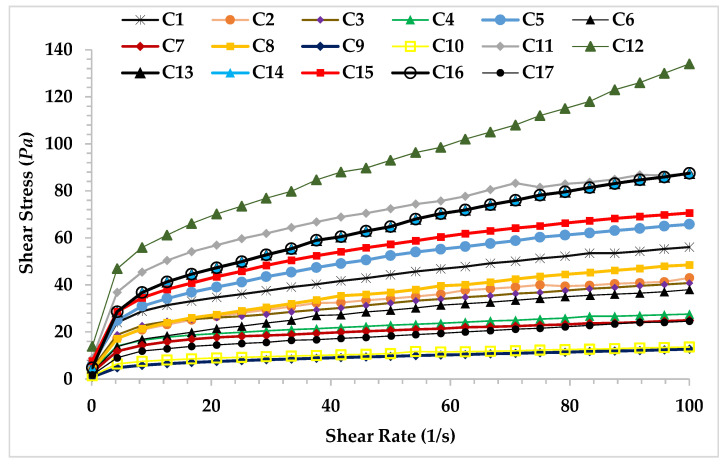
Steady shear rheological behavior of the ice cream mixes.

In this study, the data of the steady shear rheological properties were used for the formulation optimization of ice cream mixtures. The flow curves of the ice cream mix obtained from 17 different trial points are shown in Figure 1. There was a decrease in the slope of the shear rate versus shear stress graphs of the ice cream mixes, indicating that the viscosity of all samples decreased with increasing shear rate. The reduction in viscosity can be explained by the structural breakdown of the intermolecular interaction [35]. The ice cream mixes showed shear-thinning flow characteristics, which is typical flow behavior for an ice cream mix. The shear-thinning behavior is an important factor in choosing the pump size for mixing [35].

Power law model parameters (*K* and *n* values) and determination coefficient (*R*^2^) calculated for 17 different points created with the trial design are shown in Table 1. *R*^2^ values of the power law model were higher than 0.99. This shows that the power law model is suitable for determining the flow behavior properties of ice cream mixes. According to the ice cream formulations, *K* and *n* values differed and were found as 4.01–26.05 Pas^n^ and 0.23–0.38, respectively. *n* values lower than 1 indicated that all ice cream mixtures exhibited the non-Newtonian pseudoplastic flow behavior (Table 1). Dairy products generally exhibit shear-thinning (pseudoplastic) behavior with flow behavior indexes of 0 < *n* < 1 [36]. As seen in Table 1, the sample −10 (0.3% XG, 2.5% fat, 1% CSOB) showed the lowest *K* value (4.01 Pas^n^), while the sample-11 (0.4% XG, 12.5% fat, 3% CSOB) exhibited the highest *K* value (26.05 Pas^n^). The *K* value of the samples increased with increasing CSOB, gum, and fat content. These results showed that increasing the *K* value with the increase in CSOB can improve the shear-thinning properties of ice cream mixes. The desired consistency values can be obtained by using CSOB even at low-fat and gum content. Thus, CSOB can be used for improving rheological properties in low-fat ice cream.

### 3.2. The Effect of Model Parameters on K and n Value and Determination Optimum Formulation

**Figure 2:**
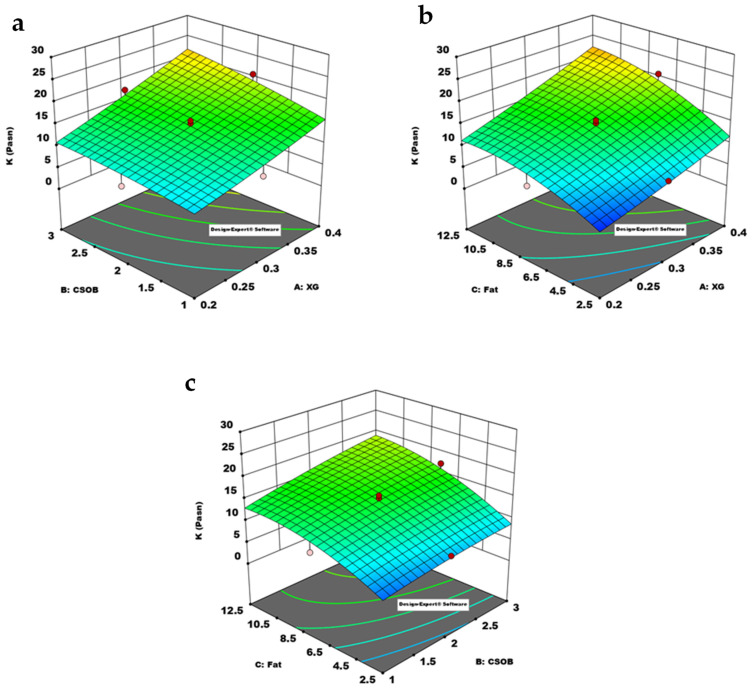
Response surface plot showing the effect of model parameters on the *K* value of ice cream mixes. (**a**): XG-CSOB, (**b**): XG-Fat, (**c**): CSOB-Fat (A: XG (xanthan gum), B: CSOB (chia seed oil by-product), C: fat (milk fat), *K*: consistency coefficient).

Figure 2 presents the impact of gum, fat, and CSOB in the formulation on *K* value. The increase in fat, gum, and CSOB, as shown in Figure 2, resulted in an increase in the *K* value of the ice cream mixes. The structure of CSOB, which contains polysaccharides with high water-holding ability, can explain this phenomenon. This polysaccharide structure offers excellent water retention and stabilizing capabilities. CSOB can be adsorbed at the interface area and has surface-active qualities, in addition to its stabilizer action. Because of these CSOB characteristics, the *K* value of the ice cream mixes increased. Furthermore, increasing the amount of gum resulted in a considerable rise in the *K* value, particularly in formulations including xanthan gum at a specific level. The increase in *K* value in both increases in fat, CSOB, and gum content can be explained by the synergistic impact of the ice cream mix’s components.

Table 2 showed that the response surface method (RSM) and the quadratic model were used to describe the influence of formulation components on the flow behavior parameters (*K*, *n*) of ice-cream mixtures. As seen in Table 2, the ANOVA analysis of variance was used to statistically evaluate the influence of dependent variables on the *K* value of the ice cream mixes. 

The *R*^2^ and Adjusted *R*^2^ values of the model used were determined as 0.9864 and 0.9618, respectively. The differences between Adjusted *R*^2^ and predicted *R*^2^ was lower than 0.2, and the lack of fit value was found as insignificant. These results indicated that the quadratic model could be successfully used to describe the effect of formulation on the *K* value of the samples. The *p*-value of the model was lower than 0.05, indicating that the model terms significantly affected the *K* value. In this model, A, B, C, AB, C² are significant model terms. The linear effect of all independent variables was significant. The interaction and model terms A and B, and the quadratic effect of C was also be found as significant.

Steady shear rheological analyzes showed that the increase in the amount of CSOB in the formulation resulted in a significant increase in the *K* values of the samples. Chia seeds affect fat binding and gel-forming properties due to the functional properties of dietary fiber. Olivos-Lugo et al. [37] reported that chia seeds are high in dietary fiber (34.6%), oil contents (32.2%), and protein (24.6%). Therewith, Akcicek and Karasu [20] suggested that CSOB could be used as a fat replacer in a low-fat salad dressing. Considering the properties of CSOB in this study, it is understood that it has a stabilizer feature due to its polysaccharide content and that the proteins it contains can be adsorbed in the interface area and have emulsifier quality. Proteins show surface-active properties and decrease the interfacial tension, which is predicted to cause an increase in consistency coefficient. The stabilizer feature comes from the branched polysaccharide structure in its content, and these polysaccharides can hold water [12]. Based on these properties, CSOB causes an increase in consistency coefficient (*K*) and can be used in the production of low-fat ice cream. With the increase in the *K* value of the mixtures, the *n* value decreases and shows pseudoplastic behavior, which is the typical flow behavior characteristic of ice cream. Due to the solid particles in CSOB, it significantly increases the *K* value by affecting the viscosity of the ice cream mixture. The fat, emulsifier, stabilizer, and CSOB used in ice cream mixtures significantly increase the *K* consistency coefficient of the mixtures. The main reason for this is the synergistic interaction between the components. The *K* value of the full-fat control sample was used to determine the optimum formulation. The sample with the highest desirability value and minimum fat content was selected as the optimum formulation. The optimum formulation was determined as 2.5% fat, 0.29% XG, 2.51% CSOB.

### 3.3. Rheological Properties of Optimum and Control Ice Cream Mixtures

**Figure 3:**
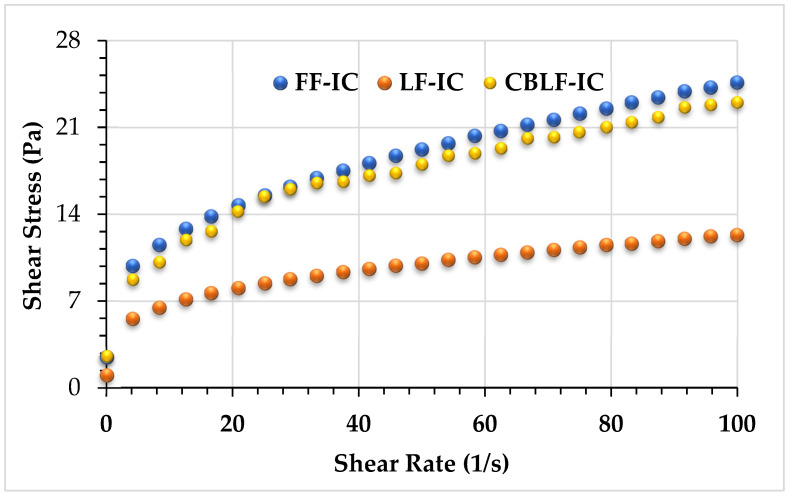
Steady shear rheological behavior of ice cream mixes. FF-IC: full-fat ice cream, LF-IC: low-fat ice cream, CBLF-IC: low-fat ice cream with chia seed oil by-product.

The steady shear, frequency sweep, and thixotropic properties of the samples produced based on optimum formulation were compared with the values of the full-fat and low-fat control sample. The flow curves of optimum and control ice cream samples were given in Figure 3. The ice cream mixtures performed a pseudoplastic (shear-thinning) rheological behavior; that is, the shear stress increased with increasing shear rate (Figure 3). Pseudoplastic rheological behavior is the typical behavior to characterize the flow properties of most ice cream mixtures [38,39]. The optimum and high-fat control samples showed similar viscosity curves. The power law model was used to determine the consistency coefficient (*K*) and flow behavior index (*n*) values of the optimal and control ice cream mixtures. Table 3 represents the power law model parameters (*K* and *n* values) as well as the determination coefficient (*R*^2^). *K* values varied from 3.46 to 5.66 Pas^n^, *n* values from 0.30 to 0.33, and *R*^2^ values higher than 0.99. While the *K* value of the full-fat control samples and the *K* value of the sample containing CSOB were found to be statistically insignificant, the *K* value of both samples was found to be significantly higher than the low-fat control. This result showed that an ice cream mix similar to the consistency properties of full-fat ice cream could be produced with the use of CSOB.

**Figure 4:**
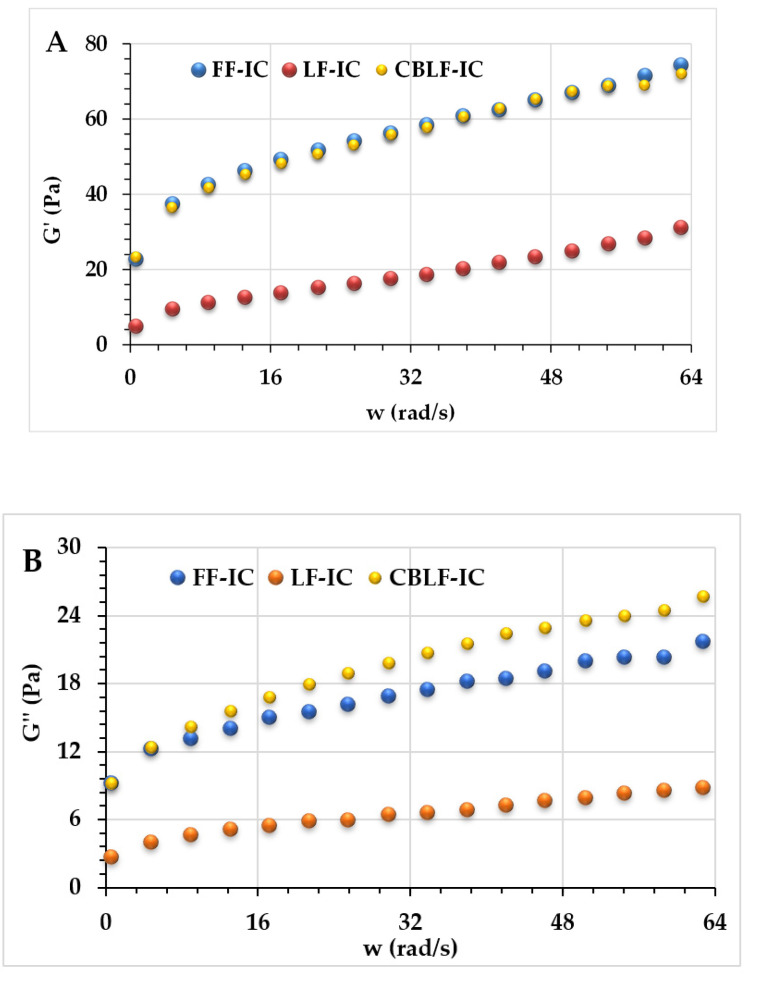
Dynamic rheological properties of the ice cream mixes. (**A**): Storage modulus ($G^{\prime }$) vs. ω, (**B**): loss modulus ($G^{\prime \prime }$) vs. ω. FF-IC: full-fat ice cream, LF-IC: low-fat ice cream, CBLF-IC: low-fat ice cream with chia seed oil by-product.

The dynamic rheological properties of ice creams produced from optimum and high-fat and low-fat control ice cream formulations were investigated. The frequency sweep test can simulate the liquid behavior of ice cream samples during chewing in the mouth [40], which helps to comprehensively evaluate the impact of CSOB addition on ice cream quality. Increasing $G^{\prime }$ and $G^{\prime \prime }$ values of samples with increasing frequency is evidence of gel-like behavior in ice cream samples [41]. As seen in Figure 4, the value of $G^{\prime }$ of all samples was higher than the value of *G*″, indicating that the solid character of all ice cream samples is more dominant than the liquid character. As seen in Figure 4, the $G^{\prime }$ value of FF-IC (12.5% fat) and CBLF-IC (2.5% fat) samples were almost at the same level, which explained that 10% fat can be compensated with 2.51% CSOB. As can be seen from the graph, the LF-IC instance has the lowest $G^{\prime }$ and the lowest $G^{\prime \prime }$. The $G^{\prime }$ and $G^{\prime \prime }$ values of the LF-IC (2.5% fat, 0.35% XG) were lower than the $G^{\prime }$ and $G^{\prime \prime }$ values of the CBLF-IC (2.5% fat, 0.29% XG, 2.51% CSOB). As it can also be seen in Table 3, the CBLF-IC has an elastic modulus value similar to FF-IC with full-fat content (12.5%). Aziz et al. [42] investigated the effect of adding okra gum on the rheological, textural, and melting properties of low-fat ice cream samples. It was stated that $G^{\prime }$ values were higher than $G^{\prime \prime }$ values for all ice cream samples. Substituting the fat content in ice cream with okra gum increased the viscous modulus ($G^{\prime \prime }$) values of the samples. Previous studies on viscoelastic properties were consistent with the results of our study in terms of $G^{\prime }$ and $G^{\prime \prime }$ values. Synergetic interactions between CSOB and ice cream ingredients can lead to improved food quality and expanded food applications due to enhanced functional properties. It may also have commercial potential for cost reduction.

The dynamic rheological parameters ($K^{\prime }$, $K^{\prime \prime }$, $n^{\prime }$, and $n^{\prime \prime }$ values) were also calculated by using the power law model (Table 3). The *R*^2^ values of the model were found in the range of 0.97–0.98. As can be seen in Table 3, the *K*′ and *K*″ values of the samples were in the range of 3.09–23.41 and 2.46–8.46, respectively; the values of *n*′ and *n*″ were found in the range of 0.268–0.535 and 0.212–0.288, respectively. The *K*′ values were higher than the *K*″ values for all samples. Accordingly, all of the ice cream samples showed a viscoelastic solid character. When CSOB was added to low-fat ice cream, the *K*′ and *K*″ values increased when compared to the low-fat ice cream sample.

**Figure 5:**
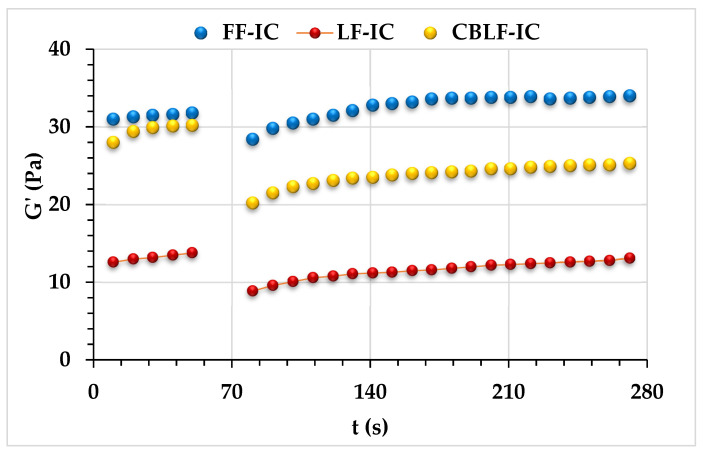
3-ITT rheological properties of ice cream mixes. FF-IC: full-fat ice cream, LF-IC: low-fat ice cream, CBLF-IC: low-fat ice cream with chia seed oil by-product.

All ice cream samples in the third interval exhibited thixotropic behavior (Figure 5). This result indicated that all ice cream samples could recover their viscoelastic character after high sudden deformation during food processing, such as homogenization or pumping. For ice cream combinations, this flow behavior is desirable. Akcicek and Karasu [20] investigated the thixotropic behavior of salad dressing samples stabilized by chia seed oil by-products and found that recoverable characteristics in the third interval are similar to our findings. The current investigation found that CSOB enhanced the thixotropic behavior of ice cream samples following rapid deformation.

Parameters (G_0_′, G_e_′, *k*, G_0_′/G_e_′) were obtained with the second-order structural kinetic model. G_0_′, G_e_′, G_0_′/G_e_′, *k* × 1000, and *R*^2^ values were 8.42–21.12, 14.81–38.73, 1.760–1.834, 11.10–27.00, and 0.995–0.996, respectively. FF-IC showed the highest G_0_′, G_e_′, G_0_′/G_e_′, and k × 1000 values, indicating that the full-fat control sample (FF-IC) showed the highest thixotropic behavior. The amount of fat in an O/W emulsion has a significant impact on its rheological characteristics. Therefore, the decrease in the fat content of the ice cream samples causes weak rheological properties, especially the recoverable character, as the fat content of the ice cream samples decreases. Although CBLF-IC has low-fat content (2.5%), FF-IC and CBLF-IC showed similar thixotropic behavior and viscoelastic solid character so the higher recoverable behavior obtained with CSOB addition can be explained by more intermolecular interactions by the formation of small aggregates of hydrocolloid. These rheological properties indicate that CSOB can be utilized to enhance the rheological properties and thixotropy of low-fat ice cream samples.

### 3.4. Emulsion Stability and Microstructure Properties of Ice Cream Mixes

**Figure 6:**
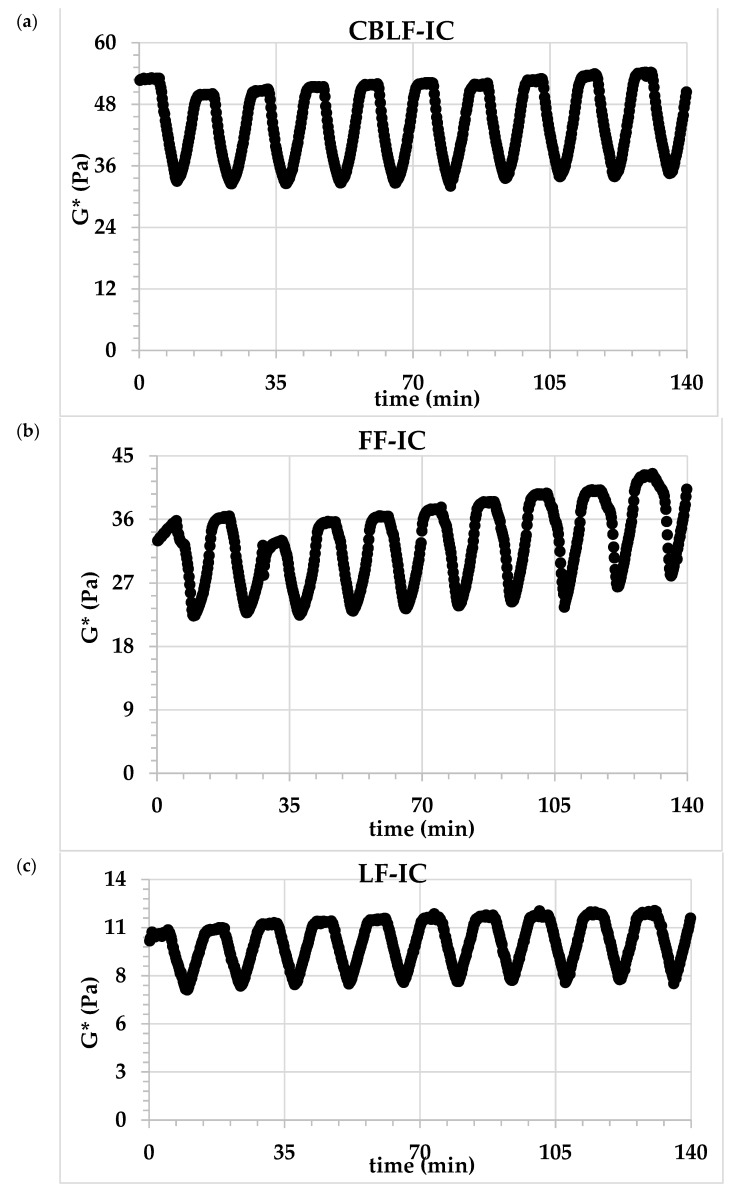
The change in the G* value after applying the thermal loop. (**a**) CBLF-IC: low-fat ice cream with chia seed oil by-product, (**b**) FF-IC: full-fat ice cream, (**c**) LF-IC: low-fat ice cream.

The emulsion stability of the ice cream mixes was determined by the thermal loop test. The thermal loop test could be used as a fast method to measure emulsion stability by subjecting to different numbers of thermal cycles. The temperature fluctuations during processing, production, storage, and transportation stages were simulated in this test [31]. The structural or morphological changes due to the applied thermal stress are determined by the change of modules (G*, *G’*) from cycle to cycle. Figure 6 shows the change in the G* value after applied thermal stress. As can be seen, after 10 applied thermal cycles, a slight increase in the G* value of all samples was observed. This insignificant change indicates that all samples are resistant to thermal stress and have high emulsion stability. The percent change (ΔG*) in the G* values of the samples was calculated as 14.37, 10.10, 8.37 for FF-IC, LF-IC, and CBLF-IC, respectively. This may indicate that the sample containing CSOB may show higher stability than other samples.

**Figure 7:**
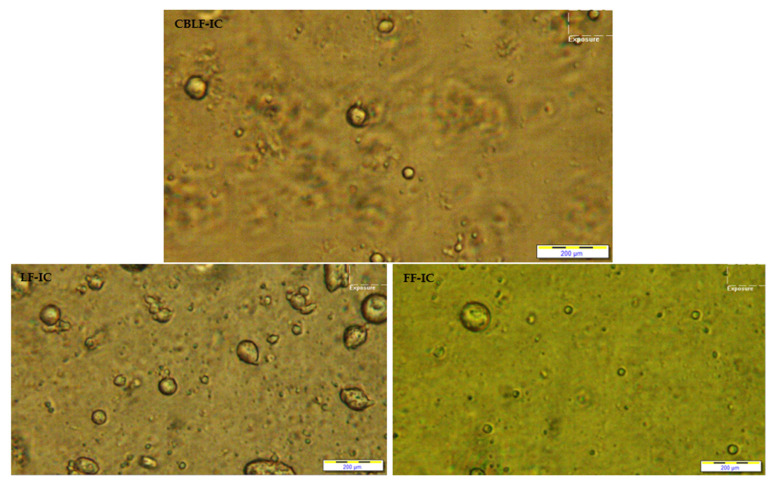
Light microscopy pictures of the ice cream mixtures. FF-IC: full-fat ice cream, LF-IC: low-fat ice cream, CBLF-IC: low-fat ice cream with chia seed oil by-product. Samples were characterized at room temperature at 20× magnification.

The fat particle size (d_32_), PDI value, and zeta potential values of the samples were found as 0.494–1.305 μm, (−39.13)–(−28.90) mV and 0.493–0.741, respectively (Table 3). As can be seen, the sample containing CSOB exhibited lower particle size and PDI value. These results are consistent with the thermal loop test. A decrease was observed in the zeta potential values of the samples as the water ratio increased. However, all samples have a sufficient zeta potential value. Figure 7 shows the milk fat particle distribution. The high-fat control sample and the sample containing CSOB have homogeneous droplet distribution and low-fat droplet size. These results indicated that the use of CSOB could have a positive effect on the fat droplet size and distribution, and emulsion stability in ice cream.

### 3.5. Quality Parameters of Ice Cream

#### 3.5.1. Thermal Properties of Ice Cream

The thermal properties of the ice cream samples are presented in Table 4. The freezing points (T_f_) of FF-IC, LF-IC, and CBLF-IC were obtained as −3.66, −3.35, and −3.99 °C, respectively. The freezing points of the ice cream samples were significantly differed (*p* < 0.05). The freezing point temperature of ice cream is closely related to the serum phase concentration and the soluble biopolymer concentration. Generally, the T_f_ value decreases as the serum phase concentration increases or the molecular weight of the soluble biopolymers decreases [43,44]. Soukoulis, Lebesi and Tzia [33] investigated the effect of different fiber contents on the thermal properties of ice cream. Similar to our study, a significant decrease was observed in the T_f_ values of ice creams when 2% wheat and oat fiber were added. Researchers explained this result by increasing the serum phase concentration due to the high water holding capacity of wheat and oat fiber. They reported an increase in the T_f_ value with the addition of apple fiber and inulin, which have higher soluble fiber content. The high water holding capacity of the insoluble fibers in the CSBO content may have caused a decrease in the T_f_ value. In addition, the use of less XG in the sample containing CSOB may have caused a decrease in the less soluble biopolymer substance in the serum phase, thus significantly reducing the T_f_ value. The higher T_f_ value obtained from LF-IC could be due to a decrease in solid concentration by reducing fat contents.

The melting resistance of ice cream represents the ability of ice cream to resist melting when exposed to high temperatures. The heating system in the DSC provides the temperature that causes the formation of an endothermic peak. The melting enthalpy (ΔH) is calculated by integrating with the area of the endothermic peak and is the amount of energy leaving the system. It occurs as a change in the overall internal energy of food [45]. In our study, the melting enthalpy values of the samples were found as 166.0, 199.3, and 146.8 J/g for the FF-IC, LF-IC, and CBLF-IC, respectively. The freezable water amount is a critical parameter affecting ice melting enthalpy in ice cream. Melting enthalpy is less in ice creams with lower freezable water content [46]. The enthalpy value of the sample CBLF-IC (containing CSOB) was found to be lower than that of FF-IC and LF-IC (the full-fat and low-fat control samples, respectively). These results can be explained by the interaction between water and CSOB. The addition of CSOB to ice creams mix could increase the amount of bound water and reduced the amount of freezable water thanks to its water-binding capacity, which may lead to a decrease in enthalpy. In addition, as the fat reduction in ice cream samples was balanced by adding water, it caused high ice formation in ice cream. However, the CSOB produced a balanced effect by chemically binding the free water, preventing excessive ice crystal formation. The lower enthalpy value of ice cream containing CSOB than FF-IC despite higher water content could be explained by the higher water retention capacity and lowering amount of freezable water.

ΔT values were found as 14.16, 15.95, and 17.05 for the samples CBLF-IC, LF-IC, and FF-IC, respectively. The sample containing CSOB had the lowest ΔT value than control samples. The temperature range (ΔT) could be used as an indicator of the uniformity of the size distribution of ice crystals. Therefore, a narrow melting temperature range indicates a more homogeneous distribution of ice crystals melting in a smaller temperature range [47]. The enrichment of ice creams in terms of polysaccharides and protein with CSOB facilitates the formation of tiny ice crystals, contributing to the improvement of texture perception and stability of ice crystals during cold storage.

#### 3.5.2. Overrun Properties of Ice Cream

The overrun values are shown in Table 4. There was a significant difference between the overrun values of the samples. The sample containing CSOB exhibited the highest overrun value, while the low-fat control sample exhibited the lowest overrun value. The increase in the overrun value with the addition of CSOB may be due to the protein and high molecular carbohydrates in the CSOB content. Proteins play an important role in increasing foam stability thanks to their emulsification properties [48]. With the addition of CSOB to the formulation, both the protein content is gained, and the high molecular carbohydrate ratio is increased. Thus, an increase in the overrun value was observed with the addition of CSOB. Akalın et al. [49] reported that dietary fibers obtained from orange, apple, and wheat provided a significant increase in overrun values compared to the control sample. On the other hand, Mansour et al. [50] reported that the addition of datary fiber powder significantly reduced the overrun value of the ice cream samples. Some researchers have suggested that there is a relationship between the overrun value and the rheological properties [6,26]. Samakradhamrongthai et al. [51] reported that the overrun value increased with increased ice cream mix viscosity. The higher overrun with increasing viscosity could be explained by a more efficient breakdown of the incorporated air cells to a smaller air cell size of ice cream mix during freezing [51,52]. Similarly, the increase in the overrun value with the addition of CSOB in our study can be explained by the increase in the consistency index of the ice cream mix.

#### 3.5.3. Sensory and Color Quality of Ice Cream

Sensory scores of ice cream samples are shown in Table 5. As can be seen, a significant difference was observed between the sensory scores of FF-IC and LF-IC. Whole-fat ice cream showed the highest value in all criteria. Low-fat ice cream containing CSOB (CBLF-IC), on the other hand, showed similar sensory properties to the full-fat sample (FF-IC), except for the color and appearance criteria. With the addition of CSOB, the change in color values compared to the full-fat sample (FF-IC) is expected. There was no significant difference in the overall acceptability criteria of the CSOB-containing sample (CBLF-IC) and the full-fat control sample (FF-IC). This indicated that the color difference observed with the addition of CSOB could not adversely affect the consumability of ice cream. Eskandari and Akbari [53] and Akın et al. [54] reported that the addition of dietary fiber and other fat replacers did not cause a negative change in sensory scores of ice cream, similar to our study. In another study [51], the reduced fat ice cream prepared with inulin showed an acceptable sensory score, similar to our study. These results indicated that with the addition of CSOB, the tested quality properties of ice cream could be improved without adversely affecting the sensory properties and that low-fat ice cream could be produced in a similar way to achieve the quality properties of full-fat ice cream. L*, a* and b* color values of the samples are presented in Table 6. L*, a* and b* values of the samples were found as 77.08–83.42, 4.23–4.93 and 13.63–15.41, respectively. As can be seen, a significant difference was detected between the color values of the samples. The highest L* value was measured from the FF-IC sample, while the lowest L* value was obtained from the CBLF-IC sample. The high L* value of the FF-IC sample can be explained by the higher fat content than the other two samples. In addition, with the introduction of CSOB into the ice cream formulation, a decrease in L*, a* and b* values was observed. This result shows that CBLF-IC will cause a significant change in the color values of the ice cream samples.

## 4. Conclusions

The consumption of chia seed oil has been increasing in recent years due to its polyunsaturated fatty acids, especially linolenic acid. As a result of the production of cold- pressed chia seed oil, waste rich in carbohydrates, fiber and protein emerges. Evaluation of this waste is an important issue for cold press manufacturers. In this study, the potential for the use of CSOB in the production of low-fat ice cream was investigated. A significant increase was observed in the *K* value of the ice cream mix samples with the increase in CSOB in the formulation. The low-fat sample (CBLF-IC) containing CSOB, produced as a result of the optimization, showed similar rheological properties to the full-fat sample (FF-IC). Emulsion stability, fat globule size and distribution, thermal properties, and overrun analyzes indicated that the quality of ice cream mix and ice cream could be improved with the addition of CSOB. According to the results of the sensory analysis, the addition of CSOB did not cause a significant decrease in the sensory qualities of ice cream. This study has shown that CSOB can be used successfully in the production of low-fat ice cream and can provide a new perspective for the evaluation of this by-product with low economic value.

## Figures and Tables

**Table 1:** Steady shear power-law parameters of the ice cream mixtures contained CSOB with ingredient compositions.

Samples	XG (%)	Fat (%)	CSOB (%)	*K* (Pas^n^)	*n*	*R* ^2^
C1	0.30	7.5	3	17.23	0.26	1.00
C2	0.20	12.5	1	10.95	0.29	1.00
C3	0.40	2.5	3	11.61	0.27	0.99
C4	0.40	2.5	1	9.46	0.23	0.99
C5	0.30	12.5	2	14.83	0.32	1.00
C6	0.20	7.5	2	7.51	0.35	1.00
C7	0.30	2.5	2	8.28	0.24	0.99
C8	0.20	12.5	3	12.57	0.35	1.00
C9	0.30	7.5	1	9.38	0.32	1.00
C10	0.30	2.5	1	4.01	0.26	0.99
C11	0.40	12.5	3	26.05	0.28	1.00
C12	0.40	7.5	2	20.69	0.37	0.99
C13	0.30	7.5	2	15.06	0.38	1.00
C14	0.30	7.5	2	15.01	0.38	1.00
C15	0.40	12.5	1	17.52	0.30	1.00
C16	0.30	7.5	2	15.62	0.37	1.00
C17	0.20	2.5	3	5.30	0.33	0.99

XG: xanthan gum, CSOB: chia seed oil by-product, *K*: consistency coefficient, *n*: flow behavior index, *R*^2^: regression coefficient corresponding to the power law model.

**Table 2:** Quadratic model parameter’s corresponding *K* value.

Source	Sum of Squares	df	Mean Squares	F-Value	*p*-Value	
Model	529.67	9	58.85	56.29	<0.0001	significant
A-XG	280.46	1	280.46	268.24	<0.0001	
B-CSOB	86.69	1	86.69	82.91	<0.0001	
C-Fat	138.80	1	138.80	132.75	<0.0001	
AB	11.93	1	11.93	11.41	0.0118	
AC	2.29	1	2.29	2.19	0.1824	
BC	0.9184	1	0.9184	0.8783	0.3799	
A²	1.91	1	1.91	1.83	0.2183	
B²	0.0066	1	0.0066	0.0063	0.9391	
C²	7.74	1	7.74	7.41	0.0297	
Residual	7.32	7	1.05			
Lack of Fit	7.09	5	1.42	12.18	0.0776	not significant
*R* ^2^					0.9864	
Adjusted *R*^2^					0.9688	
Predicted *R*^2^					0.8918	
Adeq Precision					30.5150	

XG: xanthan gum, CSOB: chia seed oil by-product, *K*: consistency coefficient.

**Table 3:** Steady shear, dynamic, and 3-ITT rheological model parameters of FF-IC, LF-IC, and CBLF-IC.

Rheological Analysis		Samples		
		FF-IC	LF-IC	CBLF-IC
Steady shear	*K* (Pas^n^)	5.66 ± 0.07 ^a^	3.46 ± 0.03 ^b^	5.53 ± 0.09 ^a^
σ = *K* × γ*^n^*	*n*	0.32 ± 0.01 ^b^	0.36 ± 0.01 ^a^	0.30 ± 0.02 ^b^
	*R* ^2^	>0.99	>0.99	>0.99
Frequency				
$G^{\prime \prime }$ = $K^{\prime \prime }$× (ω)*^n′^*	*K* ′	23.41 ± 0.18 ^a^	3.09 ± 0.06 ^b^	23.09 ± 0.24 ^a^
	*n*′	0.268 ± 0.002 ^b^	0.535 ± 0.003 ^a^	0.268 ± 0.002 ^b^
	*R* ^2^	0.993	0.979	0.9878
				
$G^{\prime \prime }$ = $K^{\prime \prime }$ × (ω)*^n″^*	*K^″^*	8.46 ± 0.12 ^a^	2.46 ± 0.02 ^b^	8.15 ± 0.35 ^a^
	*n* ^″^	0.212 ± 0.001 ^c^	0.288 ± 0.002 ^a^	0.268 ± 0.003 ^b^
	*R* ^2^	0.979	0.983	0.978
3-ITT	G_0_′	21.12 ± 0.08 ^a^	8.42 ± 0.04 ^c^	18.86 ± 0.05 ^b^
	G_e_′	38.73 ± 0.15 ^a^	14.81 ± 0.08 ^c^	26.34 ± 0.11 ^b^
	*k*	0.027 ± 0.001 ^a^	0.011 ± 0.000 ^b^	0.025 ± 0.001 ^a^
	G_e_′/ G_0_′	1.834	1.760	1.397
	*k* × 1000	27.00	11.10	25.33
	*R* ^2^	0.983	0.995	0.996
ζ-potential (mV)		−39.13 ± 1.041 ^a^	−32.03 ± 1.464 ^b^	−28.90 ± 1.058 ^c^
d_32_ (µm)		1.305 ± 0.044 ^a^	0.877 ± 0.024 ^b^	0.494 ± 0.012 ^c^
PdI		0.741 ± 0.127 ^a^	0.493 ± 0.167 ^a^	0.534 ± 0.174 ^a^

FF-IC: full-fat ice cream, LF-IC: low-fat ice cream, CBLF-IC: low-fat ice cream with chia seed oil by-product. Different lowercase letters in the same line indicate a statistical difference (*p* < 0.05).

**Table 4:** Thermal properties of the control ice cream mixtures and optimum ice cream mixture contained CSOB.

Sample	T_0_ (°C)	T_end_ (°C)	T_f_ (°C)	ΔT (°C)	ΔH_f_ (J/g)	W_f_ (%)	Overrun (%)
FF-IC	−11.67 ± 0.06 ^C^	5.39 ± 0.04 ^A^	−3.66 ± 0.02 ^B^	17.06 ± 0.05 ^A^	166.01 ± 2.68 ^B^	49.72 ± 0.28 ^B^	75.41 ± 0.18 ^B^
LF-IC	−11.0 ± 0.15 ^B^	4.95 ± 0.01 ^B^	−3.35 ± 0.05 ^A^	15.95 ± 0.08 ^B^	199.32 ± 2.45 ^A^	59.51 ± 1.05 ^A^	71.23 ± 0.31 ^C^
CBLF-IC	−10.18 ± 0.04 ^A^	3.23 ± 0.02 ^C^	−3.98 ± 0.03 ^C^	14.16 ± 0.10 ^C^	146.85 ± 1.25 ^C^	43.95 ± 0.74 ^C^	86.31 ± 0.62 ^A^

FF-IC: full-fat ice cream, LF-IC: low-fat ice cream, CBLF-IC: low-fat ice cream with chia seed oil by-product. Different letters in the same column indicate a statistical difference (*p* < 0.05).

**Table 5:** Sensory analysis of ice cream samples.

Sample	Color and Appearance	Icy Structure and Consistency	Foreign Taste and Smell	Cream Taste	Melting Resistance	General Acceptance
FF-IC	7.90 ± 1.10 ^A^	7.01 ± 1.10 ^A^	7.20 ± 1.40 ^A^	7.00 ± 2.26 ^A^	7.15 ± 2.08 ^A^	7.44 ± 1.50 ^A^
LF-IC	6.75 ± 1.25 ^B^	5.01 ± 1.73 ^B^	6.10 ± 1.73 ^A,B^	5.70 ± 1.51 ^A^	5.13 ± 1.45 ^B^	5.72 ± 1.85 ^B^
CBLF-IC	6.21 ± 0.92 ^B^	6.00 ± 1.42 ^A,B^	5.50 ± 2.51 ^A,B^	6.40 ± 1.25 ^A^	6.11 ± 1.10 ^A,B^	6.12 ± 1.35 ^A,B^

FF-IC: full-fat ice cream, LF-IC: low-fat ice cream, CBLF-IC: low-fat ice cream with chia seed oil by-product. Different letters in the same column indicate a statistical difference (*p* < 0.05).

**Table 6:** Color properties of the control ice cream mixtures and optimum ice cream mixture containing CSOB.

Sample	L*	a*	b*
FF-IC	83.42 ± 0.23 ^A^	4.93 ± 0.02 ^A^	15.41 ± 0.08 ^A^
LF-IC	79.07 ± 0.43 ^B^	4.81 ± 0.12 ^A^	15.11 ± 0.15 ^A^
CBLF-IC	77.08 ± 0.58 ^C^	4.23 ± 0.15 ^B^	13.63 ± 0.01 ^B^

FF-IC: full-fat ice cream, LF-IC: low-fat ice cream, CBLF-IC: low-fat ice cream with chia seed oil by-product. Different letters in the same column indicate a statistical difference (*p* < 0.05).

## Data Availability

The data presented in this study are available on request from the corresponding author.
